# Prevalence of resistance and toxin genes in community-acquired and hospital-acquired methicillin-resistant *Staphylococcus aureus *clinical isolates

**DOI:** 10.22038/ijbms.2020.40260.9534

**Published:** 2020-10

**Authors:** Khaled Z. El-Baghdady, Mervat I. El-Borhamy, Hisham A. Abd El-Ghafar

**Affiliations:** 1 Microbiology Department, Faculty of Science, Ain Shams University, Cairo, Egypt; 2 Microbiology Department, Faculty of Pharmacy, Misr International University, Cairo, Egypt

**Keywords:** Community, Fem, Hospital, Integron, mecA, MRSA

## Abstract

**Objective(s):**

Methicillin-resistant *Staphylococcus aureus* (MRSA) is one of the major health hazards and became of greater public health concern since the emergence of community-acquired MRSA. This work aimed to study the prevalence of *mecA*, *femA*, *femB*, *lukS*-PV, *lukF*-PV (PVL), *int*I, and *int*II genes among community-acquired (CA) hospital-acquired (HA) MRSA to increase vigilance in the diagnosis and management of suspected infections.

**Materials and Methods:**

*S. aureus* isolates recovered from clinical samples were classified into community or hospital-acquired and tested for their antibiotic susceptibility against 19 antibiotics. All isolates were screened for *mecA*, *femA*,* femB, lukS*-PV, *lukF*-PV, *int*I, and *int*II genes. Statistical correlations were carried out.

**Results:**

Out of 338 *S. aureus* isolates, only 105 were MRSA and classified as 77 CA-MRSA and 28 HA-MRSA. *mecA* and *femA* genes were present in all HA-MRSA and CA-MRSA isolates. *femB* was found in all HA-MRSA and 93.5% of CA-MRSA isolates. PVL genes were detected in 28.6% HA-MRSA isolates and 92.2% CA-MRSA. *int*I gene was recovered from 60.7% HA-MRSA isolates and 37.7% CA-MRSA isolates while the *int*II gene recovered from only 10.7% HA-MRSA isolates and 6.5% CA-MRSA.

**Conclusion:**

The high prevalence of MRSA colonizing the groin, axilla, and nose may play a significant role in endogenous infection, re-infection, and also acts as a route for MRSA transmission. *mecA* and *femA* genes could be used as a sole and fast step for identification of MRSA, while PVL genes cannot be used as a sole stable marker for CA-MRSA identification.

## Introduction

*Staphylococcus aureus *is one of the major health hazards with global presence and impact (1). It causes a wide range of infectious diseases from mild conditions, such as soft tissue infections, to severe life-threatening debilitation, such as endocarditis. Resistance of *S. aureus *to methicillin was reported for the first time in 1961 and recently *S. aureus *was identified as a major cause of nosocomial and community-acquired infections (2, 3). MRSA has become of greater public health concern since the emergence of community-acquired MRSA (4). Many hospitals struggle with increasing rates of MRSA, which is multiple drug resistant against all beta-lactam antibiotics and other types of antibiotics. Commonly, it affects patients with a longer length of stay in the intensive care unit (ICU) especially surgical ICU, dialysis unit, burn unit, and patients with permanent indwelling catheters or per-cutaneous medical devices (5). To efficiently prevent dissemination of MRSA, rapid and reliable identification, as well as close collaboration between clinicians and microbiologists, are required (6-8). Often, applicable antibiotics for treatment of MRSA are only glycopeptides like vancomycin and teicoplanin, however, during the last few years a great problem has emerged as some strains of MRSA have displayed intermediate (VISA) or full resistance (VRSA) to vancomycin. Accordingly, new treatment options for MRSA infections including daptomycin, linezolid, tigecycline, and quinupristin/dalfopristin were used (9). In the past decades, the epidemiology of MRSA infection has changed because of the emergence of the strains acquired outside the healthcare environment named community-associated *S. aureus* (CA-MRSA) (10). Methicillin resistance in *S. aureus *is mediated by the production of an altered penicillin-binding protein, PBP2a, regulated by a mobile staphylococcal cassette chromosome mec (SSC*mec*A) gene complex. *mec*A gene located on *the S. aureus* chromosome characterizes MRSA (11, 12). Detection of the certain marker rather than *mec*A gene specific for *S. aureus* is needed to distinguish MRSA from methicillin-resistant coagulase-negative staphylococci (CoNS). In addition to *the mec*A gene; *fem*A and *fem*B (*fem*) genes, which encode proteins that influence the level of methicillin resistance of *S. aureus*, were used to differentiate between MRSA and *mec*A-positive CoNS (11-13). Genetic typing targeting the *fem*A gene (universally present in all *S. aureus*) has been used to differentiate *S. aureus* from other coagulase-positive staphylococci and *S. pseudointermedius* (14, 15). One of the important cytotoxins produced by some strains of *S. aureus* is the Panton-Valentine leukocidin (PVL), encoded by two genes, *lukS*-PV and *lukF*-PV. PVL is present in the majority of CA- MRSA isolates and rarely present in hospital isolates, therefore it is recognized as a marker of community-acquired strains (16, 17). Epidemiological data suggested that high virulence of CA-MRSA was associated with PVL genes but direct evidence of association of PVL to pathogenesis had been limited (16-18). Integrons are hereditary units for gene (*int*) capture and expression, situated in the bacterial plasmid, chromosome, or transposon, which have the capability of site-specific recombination. They can also selectively capture or remove various specific drug resistance box genes, and transfer their drug resistance genes to different strains or different bacterial genera through functions, such as transformation, transduction, and conjugation, a mechanism that accelerated the spread and dissemination of bacterial drug resistance (19). Integrons are one of acquired resistances that evolves via horizontal transfer and their existence plays an important role in mediating multidrug-resistance, thus complicating the treatment of infections (20). However, research remains focused on class I, II, and III integrons (*int*I, *int*II, and *int*III). New integron types are continuously being discovered and the number of identified integron types has increased. Although the role of *int*I is well known in the spread of antibiotic resistance genes in Gram-negative bacteria, much less is known about Gram-positive bacteria and very few studies have reported the presence of *int*I in Gram-positive bacteria, as a result, increasing antibiotic resistance mediated by integrons in Gram-positive bacteria has become a great concern in the medical field (21). Integrons have been found in Gram-positive bacteria, however, their role in drug-resistant *S. aureus* remains unclear (22).

This work aimed to study the prevalence of different resistant and virulence genes (*mec*A, *fem*A, *fem*B, *luk*S-PV, *luk*F-PV (PVL), *int*I, and *int*II) among CA/HA-MRSA isolates recovered from different clinical samples, so as to increase vigilance in the diagnosis and management of suspected infections. 

## Materials and Methods

***Patients and samples***

The cases included in this study were selected from 374 patients suffering from different infections or underwent different surgeries at the International Medical Center and Ain Shams University Hospitals in the period from January 2015 to December 2017. Total of 649 samples was collected from different clinical sites including; blood (70), endotracheal tube (38), urine (42), sputum (78), diabetic foot (6), eye (18), and wound infection (82), in addition to colonization sites: groin (105), axilla (105) and nasal swabs (105). *S. aureus* recovered from the groin, axilla, and nasal swabs of one patient are considered as one strain. The research was conducted as per the Ethical code 04-Egypt-Code-of-Medical-Ethics-Ministry-of-Health-and-Population-238/2003-part four.

***MRSA isolation and identification***

Collection of samples and specimens was carried out according to Collee *et al.* (23), bacterial isolates were recovered from the provided previous samples according to Manual of Clinical Microbiology and Laboratory Manual of Microbiology (24, 25). The collected samples were cultured on blood agar (Oxoid, UK, CM0271) and incubated at 37 ^°^C for 24 hr (23). Colonies suspected to be *S. aureus *were subcultured on mannitol salt agar selective medium (Oxoid, UK, CM0085) for detection of *S. aureus *(26). Only colonies with bright yellow color after incubation for 48 hr at 37 ^°^C that showed *S. aureus* morphology with positive coagulase, catalase, and DNAse (Oxoid, UK, CM1032) activity were selected. To detect MRSA,* S. aureus* isolates were screened by disk diffusion susceptibility test using 30 µg cefoxitin disks and streaked on Oxacillin Resistance Screening Agar Base (ORSAB) (Oxoid, UK, CM1008) incubated at 37 ^°^C for 48 hr (23-25, 27).

CA-MRSA strains were distinguished from HA-MRSA strains according to the Centers for Disease Control and Prevention (CDC) (28), where diagnosis of MRSA was made in the outpatient setting or by a culture positive for MRSA within 48 hr of admission to the hospital/healthcare. The patient has no past medical history of MRSA infection or colonization, no medical history in the past one year of hospitalization (admission to a nursing home or skilled nursing facility, dialysis, and surgery), and no permanent indwelling catheters or percutaneous medical devices.

***Antibiotics susceptibility***

All 19 antibiotic disks used in this study were purchased from (Oxoid, England). The antibiotics were Cefoxitin (FOX 30 µg), Ampicillin-sulbactam (AMS 10+10 µg), Amoxicillin-Clavulanic Acid (AUG 20+10 µg), Vancomycin (VA 30 µg), Teicoplanin (TEC 30 µg), Linezolid (LZD 30 µg), Amikacin (AK 30 µg), Trimethoprim/Sulfamethoxazole (SXT 1.25 + 23.75 µg), Ciprofloxacin (CIP 5 µg), Ofloxacin (OFX 5 µg), Erythromycin (E 15 µg), Clindamycin (DA 2 µg), Nitrofurantoin (F 300 µg), Meropenem (MEM 10 µg), Imipenem (IPM 10 µg), Cefoperazone + Sulbactam (SCF 75+30 µg), Cefuroxime (CXM 30 µg), Rifampin (RA 5 µg), and Chloramphenicol (C 30 µg). An antibiotic sensitivity test was carried out according to Bauer *et al. *(29), National Committee for Clinical and Laboratory Standards Institute (CLSI) (2017) (30). The susceptibility of all *S. aureus* isolates to vancomycin and teicoplanin was performed by MIC to differentiate vancomycin and teicoplanin susceptible isolates of *S. aureus* from intermediate isolates as per CLSI (2017) (30).

***Preservation of S. aureus strains***

All *S. aureus* isolates were stored in 1 ml brain heart infusion broth medium (Oxoid Ltd, Cambridge, CB5 8BZ, UK Code**: **CM1032), with 15 % (v/v) sterile glycerol in 2 ml sterile screw cap vials then kept at -70 °C until DNA extraction. 

***PCR and genotyping***

DNA extraction was performed by QIA amp DNA Mini Kit, (Qiagen, USA) according to the manufacturer’s instructions. DNA primers used to amplify *mec*A, *fem*A, *fem*B, *luk*S-PV, and *luk*F-PV (PVL) genes as well as *Int*I* and int*II were obtained from the Sigma Company (Table 1). Reference strains *S. aureus* ATCC 46302, *S. aureus* ATCC 39362, and *S. aureus* ATCC 64132 were used as positive control. 

PCR was performed in 25 μl containing 12.5 µl Dream Taq Mastermix (Thermoscientific), 20 pmol of each primer, and 50 ng DNA using Thermocycler (Applied Biosystem 2720). The PCR conditions were adjusted as follow: for *mec*A gene amplification, initial denaturation at 94 °C for 5 min followed by 30 cycles of amplification with 94 °C for 1 min, annealing at 50 °C for 1 min, and extension at 72 °C for 2 min, ending with a final extension step at 72 °C for 10 min. The conditions for PCR amplification of *fem*A and *fem*B were: initial denaturation at 94 °C for 5 min then 25 cycles of amplification with 94 °C for 60 seconds followed by annealing at 57 °C for 60 sec, and finally extension at 72°C for 60 sec, then the final cycle at 72 °C for 10 min. The PCR program for detection of the *lukS-*PV and *lukF-*PV (PVL) genes was: initial denaturation step for 4 min at 94 °C; 30 cycles of 15 sec at 94 °C for amplification, 30 sec at 55 °C for annealing, and 30 sec at 72 °C; and final elongation at 72 °C for 7 min. The PCR program for *Int*I and *Int*II genes was as follow: initial denaturation for 4 min at 94 °C , amplification for 45 sec at 94 °C , annealing for 45 sec at 55 °C, elongation for 55 sec at 72 °C and finally, after 30 cycles, elongation for another 8 min then 5 min at 94 °C (13, 22,31,32). PCR products were run on 0.8% (w/v) agarose gel and visualized by GelDoc. Ingenius 3.

***Statistical analysis***

Data were statistically described in terms of frequencies (number of cases) and relative frequencies (percentages). The overall statistical evaluation of different studied parameters between every two groups was done by using the Chi-square test for qualitative data using a computer software program named Statistical Package for the Social Sciences (SPSS Inc., USA, ver. 17). *P*-value of 0.05 or less was adopted as statistically significant.

The sensitivity measures the proportion of actual positives which are correctly identified as such, and the specificity measures the proportion of negatives which are correctly identified.

## Results

**Figure 1:**
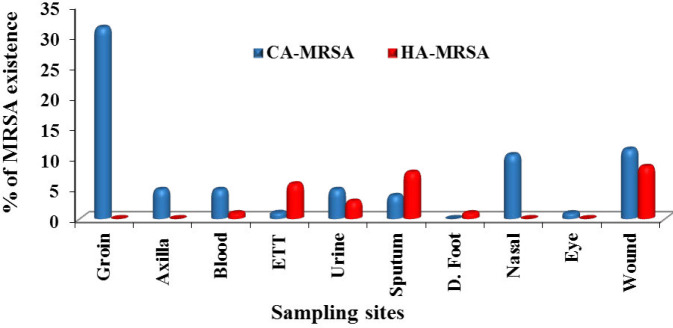
Distribution of CA-MRSA and HA-MRSA in different clinical sites. ETT: endotracheal tube

Out of 649 samples taken from 374 patients with ages ranging from 15 to 70 years (mean ≈ 42.5 years), 338 *S. aureus *isolates were recovered. Only 108 *S. aureus* isolates gave intense blue color growth on ORSAB medium after 48 hr incubation period, however, 105 of them showed resistance to 30 µg cefoxitin. The isolates that gave growth on ORSAB and were sensitive to 30 µg cefoxitin were discarded from this study. The 105 *S. aureus* (31.1%) isolates were classified as MRSA, while the rest of 338 isolates was identified as methicillin-sensitive *S*. *aureus *(MSSA). The selected (105 MRSA) were Gram-positive, coagulase, catalase, and DNAse positive. The result showed the specificity of ORSAB media for detection and identification of MRSA is 98.71% and sensitivity is 100%. According to the Centers for Disease Control and Prevention (CDC), the identified MRSA isolates were classified as CA-MRSA (77, 73.3 %) and HA-MRSA (28, 26.7%) . The distribution of CA-MRSA and HA-MRSA in different clinical samples were fully recorded and summarized (Figure 1).

**Figure 2:**
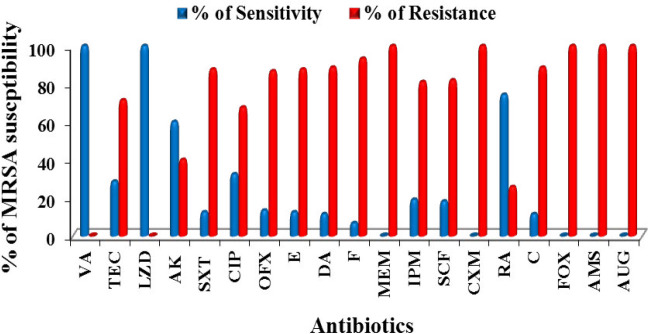
Susceptibility pattern of MRSA isolates to different antibiotics. Vancomycin (VA 30 µg), Teicoplanin (TEC 30 µg), Linezolid (LZD 30 µg), Amikacin (AK 30 µg), Trimethoprim/ Sulfamethoxazole (SXT 1.25 + 23.75 µg), Ciprofloxacin (CIP 5 µg), Ofloxacin (OFX 5 µg), Erythromycin (E 15 µg), Clindamycin (DA 2µg), Nitrofurantoin (F 300 µg), Meropenem (MEM 10 µg), Imipenem (IPM 10 µg), Cefoperazone/Sulbactam (SCF 75+30 µg), Cefuroxime (CXM 30 µg), Rifampin (RA 5µg), Chloramphenicol (C 30 µg), Cefoxitin (FOX 30 µg), Ampicillin/sulbactam (AMS 10+10 µg), Amoxicillin/Clavulanic Acid (AUG 20+10 µg)

**Figure 3:**
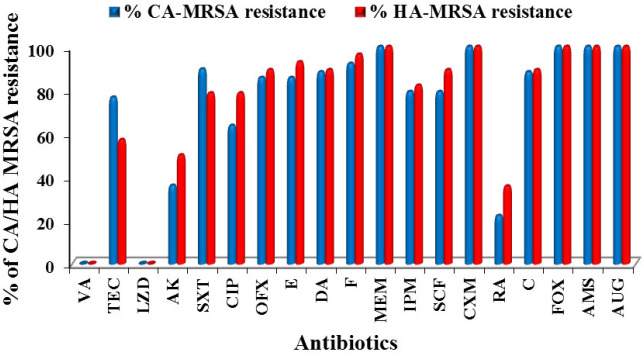
Percentage of CA- MRSA and HA-MRSA isolates resistance to tested antibiotics

The frequency of resistance and susceptibility towards 19 antibiotics with different modes of action were measured for MRSA isolates (Figure 2). All 105 MRSA isolates (100%) were sensitive to Vancomycin (MIC for all isolates ranged from 0.125 to 1 μg/ml) and Linezolid (LZD 30 µg), meanwhile, 100% degree of resistance was recorded towards Cefoxitin (FOX 30 µg), Meropenem (MEM 10 µg), Ampicillin-sulbactam (AMS 10+10 µg), Cefuroxime (CXM 30 µg), and Amoxicillin/Clavulanic Acid (AUG 20+10 µg). Percentages of CA- MRSA and HA-MRSA isolate resistances to tested antibiotics were recorded and demonstrated in Figure 3.

**Figure 4:**
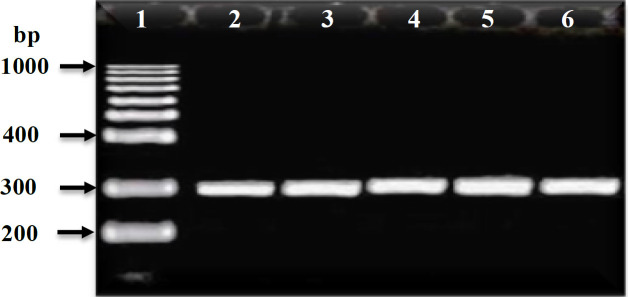
Ethidium bromide-stained agarose gel of specific PCR products of the *mec*A gene for some of HA-MRSA and CA-MRSA clinical isolates. Lane 1: 100 bp DNA marker (Promega^TM^), Lane 2: positive reference strain *S. aureus* ATCC 46302, and Lanes: 3 to 6 showed positive *mec*A gene

**Figure 5.**
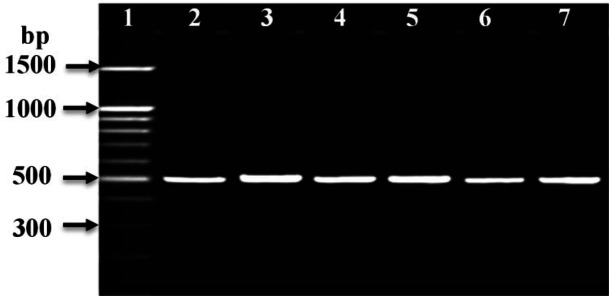
Ethidium bromide-stained agarose gel of specific PCR products of the *femA *gene for some of HA-MRSA and CA-MRSA clinical isolates. Lane 1:100 bp DNA marker (Biolabs), Lane 2: positive reference strain (*S. aureus* ATCC 46302), and Lanes: 3 to 7 showed positive *femA* gene

**Figure 6:**
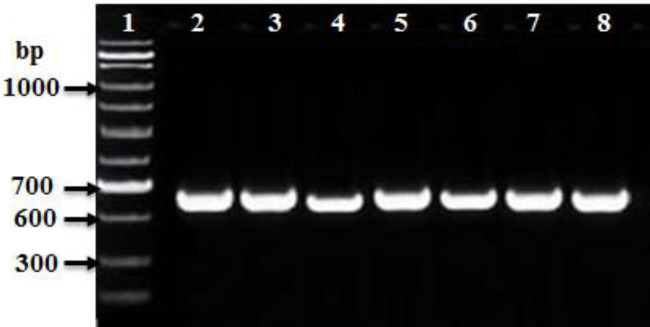
Ethidium bromide-stained agarose gel of specific PCR products of the *fem*B gene for some of HA-MRSA and CA-MRSA clinical isolates. Lane 1: 100 bp DNA marker (Genscript^R^), Lane 2: positive reference strain (*S. aureus* ATCC 46302), and Lanes: 3 to 8 showed positive* femB* gene

**Figure 7:**
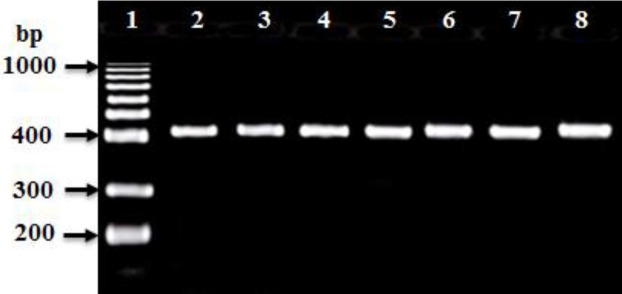
Ethidium bromide-stained agarose gel of specific PCR products of the PVL gene for some of HA-MRSA and CA-MRSA clinical isolates. Lanes 1: 100 bp DNA marker (Promega^TM^), Lane 2: positive reference strain (ATCC 46302), and Lanes: 3 to 8 showed positive PVL gene

**Figure 8:**
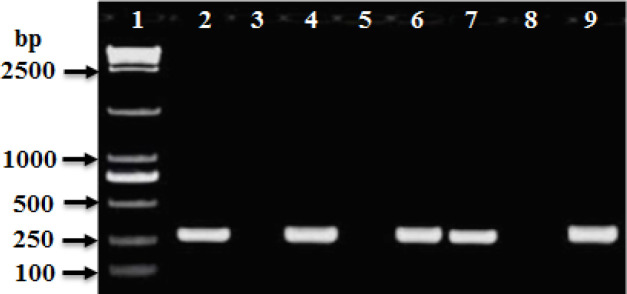
Ethidium bromide-stained agarose gel of specific PCR products of Integron I gene for some of HA-MRSA and CA-MRSA clinical isolates. Lane 1: 100 bp DNA marker (Genscript^R^ - Ready-to-UseT^TM^), Lane 2: positive reference strain (ATCC 39362), and Lanes 4, 6, 7, and 9 showed positive *int*I gene

**Figure 9:**
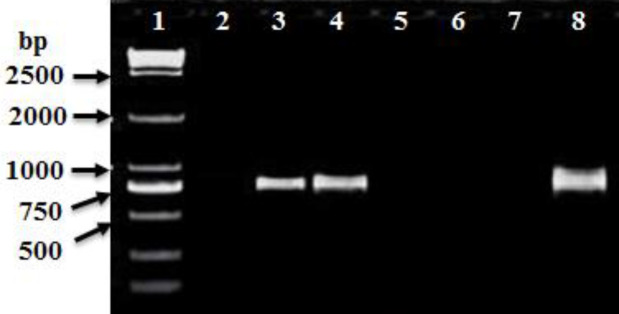
Ethidium bromide-stained agarose gel of specific PCR products of Integron II gene for some of HA-MRSA and CA-MRSA clinical isolates. Lanes 1: 100 bp DNA marker (Genscript^R^ - Ready-to-UseT^TM^), Lane 2: negative control, Lane 3: positive reference strain (ATCC 64132), and Lanes 4 and 8 showed positive *int*II gene

PCR amplification was carried out to amplify *mec*A, *fem*A, *fem*B, *luk*S-PV, and *luk*F-PV (PVL) genes and *Int*I and *int*II genes for CA-MRSA and HA-MRSA (Table 2). The PCR product for *mec*A and *fem*A genes were detected in all HA-MRSA and CA-MRSA isolates (Table 2 and Figures 4, 5). The* fem*B gene was also recovered from all HA-MRSA isolates (100%) while it was detected only in 72 (93.5%) of CA-MRSA isolates (Table 2 and Figure 6). PVL genes were recovered only from 79 MRSA strains: 8 (28.6 %) HA-MRSA and 71 (92.2%) CA-MRSA (Table 2 and Figure 7). PVL genes were detected in 90.5% of MRSA strains isolated from wound swabs: 83% from blood samples and 100 % from diabetic foot infection. Class I integron gene was recovered from 17 (60.7%) HA-MRSA isolates and 29 (37.7%) CA-MRSA isolates (Table 2 and Figure 8), while Class II gene region was observed in only 3 (10.7) HA-MRSA isolates from 5 (6.5%) CA-MRSA (Table 2 and Figure 9).

## Discussion

In the present study, the percentage of identified MRSA isolates among 338 *S*. *aureus *isolates was 31% and was statistically significant (*P*<0.05). The findings agreed with a previous surveillance study carried out to measure the antibacterial resistance in *S. aureus* and revealed the presence of methicillin resistance in 32% of *S. aureus* isolates (33). A recent study reported that methicillin resistance was observed in approximately one in three *S. aureus* isolates globally between 2004 and 2011 (34). Other surveillance studies conducted in different European countries during years 2006, 2007, and 2008 determined MRSA rate at 56.6%, 39.3%, and 42.0%, respectively (35). The present study revealed that 108 *S. aureus* isolates gave growth with intense blue color after 48 hr incubation period however the percentage of agreement between 30 µg cefoxitin susceptibility test and culturing on selective ORSAB media for detection and identification of MRSA was 97.2 %. ORSAB showed 100% sensitivity and 98.71% specificity for MRSA detection. The results of this study in agreement with earlier work indicated that ORSAB-48/hr showed 98% sensitivity and 99% specificity (36). Furthermore, another study reported that ORSAB-48 hr gave a sensitivity and specificity of 100% (37, 38). Studies conducted in 2006, demonstrated that ORSAB revealed 96% sensitivity and 99% specificity after 48 hr incubation (39, 40). In the present study, a total of 105 positive MRSA were isolated from different sites, the highest percentage, 31.4 %, was recovered from the groin (skin) followed by wound (20%), sputum (11.4%), axilla (10.5), urine (7.6%), ETT (6.7), blood (5.7%), anus (4.8) and finally 1% from diabetic foot infection and the same from eye infection. The frequency of MRSA was reported to be more in cutaneous and wound specimens (42.2%) followed by blood cultures, respiratory specimens, and urine samples (41). In this work, the highest percentage of MRSA (73.3%) was identified as CA-MRSA while (26.7%) isolates were classified as Hospital Acquired (HA) MRSA as per Centers for Disease Control and Prevention (CDC, 2007) (28), which were statistically significant *P*<0.05. High prevalence level had been also reported in a study conducted on 99 patients, 65.6% were determined to have CA-MRSA, 22.2% had CA-MSSA, 6.1% had HA-MRSA, and 6.1% had HA-MSSA (42). This finding showed high agreement with the two studies carried out in 2016 reporting that out of 139 MRSA isolates from various clinical specimens 59.7% were CA-MRSA, 35.2% were HA-MRSA, and 5% were from hospital environment (43). Similarly, it was reported (44) that from 71 MRSA isolates taken from 64 different patients admitted to the hospital, 48 (75%) had CA-MRSA while sixteen patients (25%) acquired HA-MRSA. In the present study, the antibiotic susceptibility revealed that all CA-MRSA and HA-MRSA isolates were 100% sensitive to linezolid and vancomycin only, followed by 74.6% to rifampin then amikacin 60%. The highest drug resistance rate for MRSA isolates was 100 % resistance to penicillins/β-lactam/β-lactamase inhibitor combinations (cefoxitin, ampicillin-sulbactam, cefuroxime, and amoxicillin/clavulanic acid). About 93.3% of MRSA isolates were resistant to nitrofurantoin, 88.6% of isolates were resistant to chloramphenicol, 87.6% of MRSA isolates exhibited resistance to trimethoprim/ sulfamethoxazole and erythromycin, 86.7 % to ofloxacin and clindamycin, 81.9% to cefoperazone /sulbactam then 81% to imipenem, followed by 71.4% degree of resistance toward teicoplanin, and 67.6% to ciprofloxacin which was statistically significant (*P*<0.05). This finding indicated that the phenomena of multiple drug resistance in MRSA became more and more serious. These results were consistent with those obtained by another study (45) which revealed that all MRSA isolates were 100% sensitive to vancomycin and linezolid. Similar results obtained by Ren *et al.* (22) who reported the sensitivity of MRSA to 20 types of antibiotics (penicillin, cefoxitin, oxacillin, erythromycin, clindamycin, azithromycin, bactrim, vancomycin, linezolid, amoxicillin/clavulanic acid, piperacillin/ tazobactam, ciprofloxacin, tetracycline, rifampicin, imipenem, cefazolin, cefuroxime, levofloxacin, gentamicin, and teicoplanin) were examined and the results revealed that the three antibiotics with the lowest drug resistance rate were vancomycin (0%), teicoplanin (2.2%), and (2.8%) to linezolid. 

In the present study, the HA-MRSA resistance patterns were relatively higher when compared to those of CA-MRSA except for teicoplanin and trimethoprim/ sulfamethoxazole where CA-MRSA was more resistant. Different studies supposed that both HA-MRSA and CA-MRSA possess different gene profiles like *mec*A that mediate different resistances to antibiotics and Panton-Valentine leucocidin (*PVL*) genes that cause mild skin or soft tissue infections in addition to the miss-use of antibiotics, promoting resistance that could be a possible reason for the difference in resistance patterns of HA-MRSA and CA-MRSA (46-48). The PCR products of the expected band size (310 bp) for *mec*A gene were detected in all (100%) of CA-MRSA isolates and HA-MRSA isolates based on previously published primers *mec*A gene (31). As approved by many studies all over the world; finding of *the mec*A gene is the major evidence for the detection of MRSA isolates (49-53). Contrarily, other studies reported the absence of the *mec*A gene within resistant staphylococcal isolates and moderate MRSA strains in regions worldwide with a high prevalence of MRSA which may open the door to search for other intrinsic factors that may compete with the *mec*A gene (54-59). The findings of the present study revealed that the PCR products of the expected band size (510 bp) for *fem*A gene were recovered from all CA-MRSA isolates and HA-MRSA isolates while the PCR products of the expected band size (651 bp) for *fem*B gene were recovered from all of twenty-eight (100%) HA-MRSA isolates and 72 (93.5%) out of 77 CA-MRSA isolates. This is consistent with the results obtained by another study (13) which reported that the PCR product of *fem*A and/or *fem*B was obtained from almost all the 156 MRSA strains except for five oxacillin-resistant strains (2.5%). The high expression level of *fem*A seems to be essential for high-level drug resistance MRSA (60). A study (61) was conducted on 127 highly drug resistant *S. aureus* isolates and the *fem*B gene was identified in all tested isolates (100%). Other studies were carried out during the year 2016 on a highly drug resistant *S. aureus* where the findings revealed the presence of the *fem*A gene (510 bp bands) and the *mec*A gene in all (100%) of coagulase-positive isolates (62). The findings of the present study presented that the PCR products for PVL genes were recovered from 77 out of 105 (73.3%) of MRSA isolates. Only six (21.4%) out of 28 collected HA-MRSA isolates and 71 out of 77 (92.2%) of CA-MRSA isolates were PVL gene positive. The results of this study were consistent with the previous study conducted in 2016 who reported that 90.4 % of the CA-MRSA were PVL positive, while only 4 (7.1%) of HA-MRSA strains was PVL gene positive. Studies also reported that although PVL is known as a common virulence factor of CA MRSA, 12% of HA-MRSA strains isolated from different infection sites were PVL positive (43, 63). In addition, high prevalence of the PVL gene in CA-MRSA strains was reported rather than HA-MRSA and suggested that the PVL gene may be used as an epidemiological marker of CA-MRSA infection (64). Many studies were carried out to find the correlation between the existence of the PVL gene and type of MRSA isolates during the period of 1998-2007 and the result suggested that the presence of the PVL gene represents a stable genetic marker for the CA-MRSA strains, also it may explain the frequency of primary skin infections and occasionally necrotizing pneumonia associated with these strains (65-70). A recent study was carried out to evaluate the usefulness of Panton-Valentine Leukocidin and Clindamycin susceptibility as markers of community origin of MRSA and the results revealed that both presence of *pvl* gene and susceptibility to clindamycin were found to be independent predictors of community origin of MRSA, but taken together the association was highly significant (71). In the present study, the PVL gene was recovered from 90.5% of MRSA strains isolated from wound swabs, 83% of MRSA isolated from blood samples and recovered from the strain that was isolated from diabetic foot infection (100%). This agreed with the results of a study carried on 390 MRSA isolated from a different site of infection reporting that the PVL gene positive isolates were strongly associated with skin and soft tissue diseases and these samples were four-fold more likely to contain the PVL genes compared to all other sample types (72). Furthermore, PVL positive MRSA isolates were more able to cause all types of infections than PVL negative isolates (73). All MRSA PVL gene positive strains are able to produce a pore-forming toxin that causes damage to the membrane of leukocytes leading to the destruction of white blood cells and tissue necrosis (74). In the present study, prevalence of class I and II integrons genes were carried out by the PCR method for all MRSA isolates. The expected size (280 bp) for class I integron gene was recovered from 46 out of 105 (43.8 %) MRSA isolates, divided into 17 out of 28 (60.7 %) HA-MRSA and 29 out of 77 (37.7 %) CA-MRSA isolates that showed a high level of multiple drug resistance. The expected size (788 bp) for integron class II was also recovered from only 8 (7.6%) of MRSA isolates, 3 out of 28 (10.7 %) HA-MRSA, and 5 out of 77 (6.5 %) CA-MRSA isolates that showed a degree of multiple drug resistance lower than the isolates that had the class I integron gene. The correlation between integrons class I and II *genes* detection rates in different isolates and degree of multiple drug resistance were statistically significant (*P*<0.01). These results were in agreement with the findings of the studies that suggested that prevalence of class I integrons in MRSA isolates may serve as reservoirs of multiple drug resistance and virulence-associated genes, which can contribute to the increasing rates of treatment antibiotics resistant *S. aureus* infections in both the hospital and community setting (22, 75-78). Another study was carried out by Goudarzi *et al. *(79) who reported that out of 80 multiple drug-resistant* S. aureus* isolates, Class I and II integrons were found in 56.3% and 18.7%, respectively. Another study was carried out on 106 MRSA isolates collected from burn wounds, and the result revealed the presence of class I integron in 58 (54.7%) isolates and class II integron in 3.8% of isolates. Many studies concluded that the prevalence of integrons I and II in multi-drug resistant *S. aureus* had been increased where the more drug-resistant rate *S. aureus *revealed the higher carrying rate of class I integrons (80). Moreover, the existence rate of class I integron in the plasmid is higher than the existence rate of class II integrons, and the plasmid is the main carrier for transferring integrons between multiple drug resistant *S. aureus* (22, 77,78).

**Table 1:** Primers used for PCR amplification of the studied genes

**Gene**	**Primer sequences (5'→3')**	**Size (bp)**	**Ref.**
*mec*Al-F	GTA GAA ATG ACT GAA CGT CCG ATA A	**310 **	(31)
*mec*A2-R	CCA ATT CCA CAT TGT TTC GGT CTA A		
*fem*A-F	AAAAAAGCACATAACAAGCG	**510 **	(13)
*fem*A-R	GATAAAGAAGAAACGAGCAG		
*fem*B- F	TTACAGAGTTAACTGTTACC	**651**	
*fem*B-R	ATACAAATCCAGCACGCTCT		
*Luk*PV-1F	ATCATTAGGTAAAATGTCTGGACATGATCCA	**433**	(32)
*Luk*PV-2 R	GCATCAAGTGTATTGGATAGCAAAAGC		
*Int*I F	CCTCCCGCACGATGATC	**280**	(22)
*Int*I R	TCCACGCATCGTCAGGC		
*Int*II F	GTAGCAAACGAGTGACGAAATG	**788**	
*Int*II R	CACGGATATGCGACAAAAAGGT		

F: Forward; R: Reverse

CA-MRSA: community-associated methicillin resistance *Staphylococcus aureus; *HA-MRSA: hospital-acquired methicillin resistance *Staphylococcus aureus*

MRS: Methicillin resistance *Staphylococcus aureus*

CA-MRSA: community-associated methicillin resistance *Staphylococcus aureus; *HA-MRSA: hospital-acquired methicillin resistance *Staphylococcus aureus*

CA-MRSA: community-associated methicillin resistance *Staphylococcus aureus; *HA-MRSA: hospital-acquired methicillin resistance *Staphylococcus aureus*

CA-MRSA: community-associated Methicillin resistance *Staphylococcus aureus; *HA-MRSA: hospital-acquired Methicillin resistance *Staphylococcus aureus*

CA-MRSA: community-associated methicillin resistance *Staphylococcus aureus; *HA-MRSA: hospital-acquired methicillin resistance *Staphylococcus aureus*

**Table 2:** Existence of different genes in hospital-acquired methicillin resistance *Staphylococcus aureus *(HA-MRSA) and community-associated methicillin resistance *Staphylococcus aureus *(CA-MRSA)

**Gene Type**	**HA-MRSA (28)**		**CA-MRSA (77)**	
	**No.**	**%**	**No.**	**%**
***mec*** **A**	**28**	**100**	**77**	**100**
***fem*** **A**	**28**	**100**	**77**	**100**
***fem*** **B**	**28**	**100**	**72**	**93.5**
**PVL**	**8**	**28.6**	**71**	**92.2**
***Int*** **I**	**17**	**60.7**	**29**	**37.7**
***Int*** **II**	**3**	**10.7**	**5**	**6.5**

CA-MRSA: community-associated methicillin resistance *Staphylococcus aureus; *HA-MRSA: hospital-acquired methicillin resistance *Staphylococcus aureus*

CA-MRSA: community-associated methicillin resistance *Staphylococcus aureus; *HA-MRSA: hospital-acquired methicillin resistance *Staphylococcus aureus*

## Conclusion

The high prevalence of colonized MRSA in the groin, axilla, and nose may play a significant role in endogenous infection, cross-infection, re-infection, and act as a route for MRSA transmission. The presence of *mec*A and *fem*A genes in 100% of MRSA isolates may give a recommendation to be used as a sole and fast step for identification of MRSA. The presence of PVL genes in both CA-MRSA (92.2%) and HA-MRSA (28.6 %) may give a recommendation that it cannot be used as a sole stable marker for CA-MRSA identification. The evidence of integron genes existence in some of MRSA isolates clarifies the association of its existence and the high multiple drug resistance rate recorded rather than the type of MRSA and its site of infections and/or colonization.

## References

[1] Simor AE, Goodfellow J, Louie L, Louie M (2001). Evaluation of a new medium; oxacillin resistance screening agar base, for detection of methicillin resistant Staphylococcus aureus from clin specimens. J Clin Microbiol.

[2] Cherkaoui A, Renzi G, François P, Schrenzel J (2007). Comparison of four chromogenic media for culture-based screening of meticillin-resistant Staphylococcus aureus. J Med Microbiol.

[3] Xie X, Bao Y, Ouyang N, Dai X, Pan K, Chen B, Deng Y, Wu X, Xu F, Li H, Huang S (2016). Molecular epidemiology and characteristic of virulence gene of community-acquired and hospital-acquired methicillin-resistant Staphylococcus aureus isolates in Sun Yat-sen Memorial hospital, Guangzhou, Southern China. BMC Inf Dis.

[4] Nahimana I, Francioli P, Blanc DS (2006). Evaluation of three chromogenic media (MRSA-ID, MRSA-Select and CHROMagar MRSA) and ORSAB for surveillance cultures of methicillin-resistant Staphylococcus aureus. Clin Microbiol Inf.

[5] Domann E, Hossain H, Füssle R, Chakraborty T (2000). Rapid and reliable detection of multiresistent Staphylococcus aureus (MRSA) by multiplex PCR. Dtsche Med Wochenschr.

[6] Farr BM, Jarvis WR (2002). Would active surveillance cultures help control healthcare-related methicillin-resistant Staphylococcus aureus infections?. Inf Cont Hosp Epidemiol.

[7] Badawi H, Omar M, Helmi H (2001). Evaluation of screening method for detection and typing of MRSA strains involved in noscomial spread. Eg J Med Microbiol.

[8] Cetinkol Y, Altindiş M, Cetinkaya Z, Aktepe OC (2008). Short communication: Determination of methicillin resistance in Staphylococci with different methods and detection of multiple antibiotic resistance. Mikrobiyol Bulteni.

[9] Naqao M, Okamto A, Yamada K, Haseqawa T, Haseqawa Y, Ohta M (2009). Variations in amount of TSST-1 produced by clinical methicillin resistant Staphylococcus aureus (MRSA) isolates and allelic variation in accessory gene regulator (agr) locus. J Clin Microbiol.

[10] Hsueh PR, Teng LJ, Chen WH, Pan HJ, Chen ML, Chang SC, Lin FY (2004). Increasing prevalence of methicillin-resistant Staphylococcus aureus causing nosocomial infections at a university hospital in Taiwan from 1986 to 2001. Antimicrob Agents Chemother.

[11] Francois P, Pittet D, Bento M, Pepey B, Vaudaux P, Lew D, Schrenzel J (2003). Rapid detection of methicillin-resistant Staphylococcus aureus directly from sterile or nonsterile clinical samples by a new molecular assay. J Clin Microbiol.

[12] Nozaki C, Masaki T, Kim SJ, Cruz RS, Bermido CM, Kim KY, Park C (2015). Comparative prevalence of community-acquired-methicillin-resistant Staphyloccocus aureus (CA-MRSA) among students of Centro Escolar University (Philippines), Kumamoto Health Science University (Japan) and Daegu Health College (Korea). Biomed Res.

[13] Paniagua-Contreras G, Sáinz-Espuñes T, Monroy-Pérez E, Rodríguez-Moctezuma JR, Arenas-Aranda D, Negrete-Abascal E, Vaca S (2012). Virulence markers in Staphylococcus aureus strains isolated from hemodialysis catheters of Mexican patients. Adv Microbiol.

[14] Li X, Xiong Y, Fan X, Zhong Z, Feng P, Tang H, Zhou T (2008). A study of the regulating gene of femA from methicillin-resistant Staphylococcus aureus clin isolates. J Int Med Res.

[15] Ishihara K, Shimokubo N, Sakagami A, Ueno H, Muramatsu Y, Kadosawa TY, anagisawa C, Hanaki H, Nakajima C, Suzuki Y, Tamura Y (2010). Occurrence and molecular characteristics of methicillin-resistant Staphylococcus aureus and methicillin-resistant Staphylococcus pseudintermedius in an academic veterinary hospital. Appl Env Microbiol.

[16] Vandenesch F, Naimi T, Enright MC, Lina G, Nimmo GR, Heffernan H (2003). Community-acquired methicillin-resistant Staphylococcus aureus carrying Panton-Valentine leukocidin genes: worldwide emergence. Emerg Infec Dis.

[17] Genestier AL, Michallet MC, Prévost G, Bellot G, Chalabreysse L, Peyrol S, Vandenesch F (2005). Staphylococcus aureus Panton-Valentine leukocidin directly targets mitochondria and induces Bax-independent apoptosis of human neutrophils. J Clin Invest.

[18] Li M, Cheung GY, Hu J, Wang D, Joo HS, DeLeo FR, Otto M (2010). Comparative analysis of virulence and toxin expression of global community-associated methicillin-resistant Staphylococcus aureus strains. J Inf Dis.

[19] Nield BS, Holmes AJ, Gillings MR, Recchia GD, Mabbutt BC, Nevalainen KH, Stokes, H W (2001). Recovery of new integron classes from environmental DNA. FEMS Microbiol Lett.

[20] Deng Y, Liu J, Peters BM, Chen L, Miao J, Li B, Shirtliff ME (2015). Antimicrobial resistance investigation on Staphylococcus strains in a local hospital in Guangzhou, China, 2001–2010. Microbial Drug Resist.

[21] Xu Z, Li L, Alam MJ, Zhang L, Yamasaki S, Shi L (2008). First confirmation of integron-bearing methicillin-resistant Staphylococcus aureus. Current Microbiol.

[22] Ren C, Zhao Y, Shen Y (2013). Analysis of the effect of integrons on drug-resistant Staphylococcus aureus by multiplex PCR detection. Mol Med Rep.

[23] Collee JG, Miles RS, Watt B (1996). Tests for identification of bacteria. Mackie and McCartney Practical Medical Microbiology.

[24] Murray PR, Baron EJ, Pfaller MA, Tenover FC, Yolken RH (1999). American Society for Microbiol, Manual of Clinical Microbiolog.

[25] Cappuccino JG, Sherman N (2004). Microbiol, Laboratory manual Person education. INC New Delhi.

[26] Koneman EW, Allen SD, Janda WM, Schreckenberger PC, Winn-Jr WC (1992). The gram-positive cocci part II: Streptococci and Streptococcus-like bacteria. Color Atlas and Textbook of Diagnostic Microbiology 4th Edition Philadelphia, USA: JB Lippincott.

[27] Clinical and Laboratory Standards Institute (2012). Performance Standards for Antimicrobial Susceptibility Testing of Anaerobic Bacteria.

[28] Siegel JD, Rhinehart E, Jackson M, Chiarello L (2007). Guideline for isolation precautions: Preventing transmission of infectious agents in healthcare settings.

[29] Bauer AW, Kirby WM, Sherris JC, Turck M (1966). Antibiotic susceptibility testing by a standardized single disk method. Am J Clin Pathol.

[30] National Committee for Clinical Laboratory Standards (2017). Antimicrobial susceptibility testing. National Committee for Clinical Laboratory Standards (NCCLS).

[31] Zhang K, Sparling J, Chow BL, Elsayed S, Hussain Z, Church DL, Gregson DB, Louie T, Conly JM (2004). New quadriplex PCR assay for detection of methicillin and mupirocin resistance and simultaneous discrimination of Staphylococcus aureus from coagulase-negative staphylococci. J Clin Microbiol.

[32] Lina G, Piémont Y, Godail-Gamot F, Bes M, Peter MO, Gauduchon V, Vandenesch F, Etienne J (1999). Involvement of Panton-Valentine leukocidin-producing Staphylococcus aureus in primary skin infections and pneumonia. Clin Inf Dis.

[33] Udo EE, Al-Sweih N, Dhar R, Dimitrov TS, Mokaddas EM, Johny M, Al-Obaid IA, Gomaa HH, Mobasher LA, Rotimi VO, Al-Asar A (2008). Surveillance of antibacterial resistance in Staphylococcus aureus isolated in Kuwaiti hospitals. Med Principles Practice.

[34] Festus T, Mukesi M, Moyo SR (2016). The distribution of methicillin resistant Staphylococcus aureus isolated at the Namibia Institute of Pathology in WindHoek Namibia. Ind J Med Res Pharm Sci.

[35] Haznedaroğlu T, Öncül O, Hoşbul T, Çavuşlu Ş, Baylan O, Özyurt M (2010). Yatan hastalardan soyutlanan Staphylococcus aureus suşlarında metisilin direnci: Üç Yıllık Trend, TAF. Prev Med Bull.

[36] Becker A, Forster DH, Kniehl E (2002). Oxacillin resistance screening agar base for detection of methicillin-resistant Staphylococcus aureus. J Clin Microbiol.

[37] Zeeshan M, Jabeen K, Khan E, Irfan S, Ibrahim S, Parween Z, Zafar A (2007). Comparison of different phenotypic methods of detection of methicillin resistance in Staphylococcus aureus with the molecular detection of mecA gene. J Coll Phys Surg Pak.

[38] Stoakes L, Reyes R, Daniel J, Lennox G, John MA, Lannigan R, Hussain Z (2006). Prospective comparison of a new chromogenic medium, MRSA Select, to CHROM agar MRSA and mannitol-salt medium supplemented with oxacillin or cefoxitin for detection of MRSA. J Clin Microbiol.

[39] Ngoyen V, Kitzis J, Chalfine A, Carlet A, Ben A, Goldstein F (2006). Detection of nasal colonization MRSA: a prospective study comparing real-time genetic amplification assay vs selective chromogenic media. Path Biologie.

[40] Nsira SB, Dupuis M, Leclercq R (2006). Evaluation of MRSA Select, a new chromogenic medium for the detection of nasal carriage of methicillin-resistant Staphylococcus aureus. Int J Antimicrob Agents.

[41] Tiwari HK, Sapkota D, Sen MR (2008). High prevalence of multidrug-resistant MRSA in a tertiary care hospital of northern India. Inf Drug Resist.

[42] Yang ES, Tan J, Eells S, Rieg G, Tagudar G, Miller LG (2010). Body site colonization in patients with community-associated methicillin-resistant Staphylococcus aureus and other types of S aureus skin infections. Clin Microbiol and Inf.

[43] Bhatta DR, Cavaco LM, Nath G, Kumar K, Gaur A, Gokhale S, Bhatta DR (2016). Association of Panton Valentine leukocidin (PVL) genes with methicillin resistant Staphylococcus aureus (MRSA) in Western Nepal: a matter of concern for community infections (a hospital based prospective study). BMC Inf Dis.

[44] Abdallah, SA, Al-Asfoor KK, Salama, MF, Al-Awadi BM (2013). Prospective analysis methicillin-resistant Staphylococcus aureus and its risk factors. J Global Inf Dis.

[45] Abbas A, Nirwan PS, Srivastava P (2015). Prevalence and antibiogram of hospital acquired-methicillin resistant Staphylococcus aureus and community acquired-methicillin resistant Staphylococcus aureus at a tertiary care hospital National Institute of Medical Sciences. Community Acquir Inf.

[46] Stevenson KB, Searle K, Stoddard G, Samore MH (2005). Methicillin-resistant Staphylococcus aureus and vancomycin-resistant enterococci in rural communities, western United States. Emerg Infec Dis.

[47] Huang H, Flynn NM, King JH, Monchaud C, Morita M, Cohen SH (2006). Comparisons of community-associated methicillin-resistant Staphylococcus aureus (MRSA) and hospital-associated MSRA infections in Sacramento, California. J Clin Microbiol.

[48] Vysakh PR, Jeya M (2013). A comparative analysis of community acquired and hospital acquired methicillin resistant Staphylococcus aureus. J Clin Diag Res.

[49] Murakami K, Minamide W, Wada K, Nakamura E, Teraoka H, Watanabe S (1991). Identification of methicillin-resistant strains of staphylococci by polymerase chain reaction. J Clin Microbiol.

[50] Sharma VK, Hackbarth CJ, Dickinson TM, Archer GL (1998). Interaction of native and mutant mecI repressors with sequences that regulate mecA, the gene encoding penicillin-binding protein 2a in methicillin-resistant staphylococci. J Bacteriol.

[51] Cloney L, Marlowe C, Wong A, Chow R, Bryan R (1999). Rapid detection of mecA in methicillin resistant Staphylococcus aureus using Cycling Probe Technology. Molec Cell Probes.

[52] Wongwanich S, Tishyadhigama P, Paisomboon S, Ohta T, Hayashi H (2000). Epidemiological analysis of methicillin resistant Staphylococcus aureus in Thailand. Southeast Asian J Trop Med Public Health.

[53] Hafez EE, Al-Sohaimy SA, El-Saadani MA (2009). The effect of the mecA gene and its mutant form on the response of S aureus to the most common antibiotics. Int J Immunol Stud.

[54] Chambers HF, Archer G, Matsuhashi M (1989). Low-level methicillin resistance in strains of Staphylococcus aureus. Antimicrob Agents Chemother.

[55] Ba X, Harrison EM, Edwards GF, Holden MT, Larsen, AR, Petersen A, Holmes MA (2013). Novel mutations in penicillin-binding protein genes in clin Staphylococcus aureus isolates that are methicillin resistant on susceptibility testing but lack the mec gene. J Antimicrob Chemother.

[56] Aziz HW, Al-Dulaimi TH, Al-Marzoqi AH, Ahmed NK (2014). Phenotypic detection of resistance in Staphylococcus aureus isolates: Detection of (mecA and femA) gene in methicillin resistant Staphylococcus aureus (MRSA) by Polymerase Chain Reaction. J Nat Sci Res.

[57] Elhassan MM, Ozbak HA, Hemeg HA, Elmekki MA, Ahmed LM (2015). Absence of the mecA gene in methicillin resistant Staphylococcus aureus isolated from different clinical specimens in Shendi city, Sudan. BioMed Res Int.

[58] Ligozzi M, Rossolini GM, Tonin EA, Fontana R (1991). Nonradioactive DNA probe for detection of gene for methicillin resistance in Staphylococcus aureus. Antimicrob Agents Chemother.

[59] Hiramatsu K, Kihara H, Yokota T (1992). Analysis of borderline-resistant strains of methicillin-resistant Staphylococcus aureus using polymerase chain reaction. Microbiol Immunol.

[60] Li M, Du X, Villaruz AE, Diep BA, Wang D, Song Y, Tian Y, Hu J, Yu F, Lu Y, Otto M, Otto M (2012). MRSA epidemic linked to a quickly spreading colonization and virulence determinant. Nat Med.

[61] Pournajaf A, Ardebili A, Allah-Ghaemi E, Omidi S, Borhani K, Khodabandeh M, Davarpanah M (2015). Identification of clinical methicillin and mupirocin-resistant Staphylococcus aureus by multiplex-PCR. J Med Bacteriol.

[62] Rostamzad A, Rostamneia N (2016). Prevalence of the Panton-Valentine leukocidin gene in clin isolates of Staphylococcus aureus isolated from hospitals the Ilam Province of Iran. Avicenna. J Clin Microbiol Inf.

[63] Özekinci T, Dal T, Yanık K, Özcan N, Can Ş, Tekin A, Yıldırım HI, Kandemir I (2014). Panton-Valentine leukocidin in community and hospital-acquired Staphylococcus aureus strains. Biotechnol Biotechnol Equip.

[64] Nichol KA, Adam HJ, Roscoe DL, Golding GR, Lagacé-Wiens PR, Hoban DJ (2013). Antimicrobial Resistance Alliance (CARA), Zhanel GG, Hoban DJ, Adam HJ. Changing epidemiology of methicillin-resistant Staphylococcus aureus in Canada. J Antimicrob Chemother.

[65] Collignon P, Gosbell I, Vickery A, Nimmo G, Stylianopoulos T, Gottlieb T (1989). Community-acquired meticillin-resistant Staphylococcus aureus in Australia. Lancet.

[66] Herold BC, Immergluck LC, Maranan MC, Lauderdale DS, Gaskin RE, Boyle-Vavra S (1998). Community-acquired methicillin-resistant Staphylococcus aureus in children with no identified predisposing risk. JAMA.

[67] Naimi TS, LeDell KH, Boxrud DJ, Groom AV, Steward CD, Johnson SK, Osterholm MT (2001). Epidemiology and clonality of community-acquired methicillin-resistant Staphylococcus aureus in Minnesota, 1996–1998. Clin Inf Dis.

[68] Dufour P, Gillet Y, Bes M, Lina G, Vandenesch F, Floret D, Richet H (2002). Community-acquired methicillin-resistant Staphylococcus aureus infections in France: emergence of a single clone that produces Panton-Valentine leukocidin. Clin Inf Dis.

[69] Gillet Y, Issartel B, Vanhems P, Fournet JC, Lina G, Bes M, Etienne J (2002). Association between Staphylococcus aureus strains carrying gene for Panton-Valentine leukocidin and highly lethal necrotising pneumonia in young immunocompetent patients. Lancet.

[70] Boyle-Vavra S, Daum RS (2007). Community-acquired methicillin-resistant Staphylococcus aureus: the role of Panton–Valentine leukocidin. Lab Investig.

[71] Shashindran N, Nagasundaram N, Thappa DM, Sistla S (2016). Can Panton Valentine Leukocidin gene and clindamycin susceptibility serve as predictors of community origin of MRSA from skin and soft tissue infections?. J Clin Diagn Res.

[72] Shallcross LJ, Williams K, Hopkins S, Aldridge RW, Johnson AM, Hayward AC (2010). Panton–Valentine leukocidin associated staphylococcal disease: a cross-sectional study at a London hospital, England. Clin Microbiol Inf.

[73] Cupane L, Pugacova N, Berzina D, Cauce V, Gardovska D, Miklaševics E (2012). Patients with Panton-Valentine leukocidin positive Staphylococcus aureus infections run an increased risk of longer hospitalization. Int J Molec Epidemiol and Genet.

[74] Ambrozova, H, Maresova V, Fajt M, Pavlicek P, Rohacova H, Machova I, Petras P (2013). The first case of fatal pneumonia caused by Panton–Valentine leukocidin-producing Staphylococcus aureus in an infant in the Czech Republic. Folia Microbiol.

[75] Madiyarov RS, Bektemirov AM, Ibadova GA, Abdukhalilova GK, Khodiev AV, Bodhidatta L (2010). Antimicrobial resistance patterns and prevalence of class 1 and 2 integrons in Shigella flexneri and Shigella sonnei isolated in Uzbekistan. Gut Pathogens.

[76] Marathe NP, Nagarkar SS, Vaishampayan AA, Rasane MH, Samant SA, Dohe V (2015). High prevalence of class 1 integrons in clin isolates of methicillin-resistant Staphylococcus aureus from India. Ind J Med Microbiol.

[77] Xu Z, Li L, Shi L, Shirtliff ME (2011a). Class 1 integron in staphylococci. Molec Biol Rep.

[78] Xu Z, Li L, Shirtliff ME, Peters BM, Li B, Peng Y (2011b). Resistance class 1 integron in clin methicillin resistant Staphylococcus aureus strains in southern China, 2001–2006. Clin Microbiol Inf.

[79] Goudarzi M, Seyedjavadi SS, Azad M, Goudarzi H, Azimi H (2016). Distribution of spa types, integrons and associated gene cassettes in Staphylococcus aureus strains isolated from intensive care units of hospitals in Tehran, Iran. Arch Clin Inf Dis.

[80] Goudarzi M, Seyedjavadi SS, Nasiri MJ, Goudarzi H, Nia RS, Dabiri H (2017). Molecular characteristics of methicillin-resistant Staphylococcus aureus (MRSA) strains isolated from patients with bacteremia based on MLST, SCCmec, spa, and agr locus types analysis. Microbial Path.

